# Research on the influence of external water vapor generation module on vacuum sublimation-rehydration thawing batch chicken breasts

**DOI:** 10.1016/j.fochx.2026.104400

**Published:** 2026-09-01

**Authors:** Yuyao Sun, Shanshan Chen, Weidong Wu, Nating Xu, Fangran Liu, Anyuan Xue

**Affiliations:** a School of Energy and Power Engineering, University of Shanghai for Science and Technology, Shanghai 200093, China; b Vehicle Energy and Safety Laboratory, Department of Mechanical Engineering, Ningbo University of Technology, Ningbo 315336, PR China

**Keywords:** Vacuum sublimation-rehydration thawing, External water vapor generation module, Rehydration temperature, Water vapor utilization ratio, Batch chicken breast, Batch thawing

## Abstract

This study optimized vacuum sublimation-rehydration thawing (VSRT) using an external water vapor generation module (EWVGM) for batch frozen chicken breast processing. The influences of different rehydration temperatures on batch thawing uniformity, thawing effect, thawing efficiency, energy consumption, and rehydration effect were researched. Moderately increasing the rehydration temperature enhanced mass and heat transfer, causing the uniform and quick ice crystal melting. A rehydration temperature of 50 °C was optimal, obtaining the shortest thawing time (80.08 min), the best batch thawing uniformity (with a temperature coefficient of variation of 2.16 × 10^−2^), the lowest thawing loss (1.15%), the smallest ΔpH (0.01), the lowest ΔE (2.31), the best texture parameters, and the lowest total specific energy consumption (1.465 MJ/kg). Under this thawing condition, with only 158 g of water consumed, the process achieved the highest moisture change ratio (1.85%) and the highest water vapor utilization ratio (23.42%). Compared with air thawing, vacuum steam thawing and VSRT (with internal water vapor generation), VSRT with EWVGM shortened 67.86%, 45.43%, and 14.05% of thawing time, and reduced the temperature coefficient of variation by 78.23%, 72.83%, and 22.02%, respectively. Meanwhile, VSRT with EWVGM obtained the greatest thawing effects, the best energy saving performance, and the highest water vapor utilization ratio. This provided guidance for batch frozen food thawing.

## Highlights

• External water vapor generation is used in vacuum sublimation-rehydration thawing.
• The optimized system is suitable for batch thawing of chicken breasts.
• The thawing condition at rehydration temperature of 50 °C is the best.
• The thawing efficiency and effect are superior to that of vacuum steam thawing.
• The consumption of electricity and water is less than that of vacuum steam thawing.

## 1 Introduction

The quality of chicken breast is high, mainly in low in fat and calories yet rich in protein, and relatively inexpensive. Consequently, it is widely favored by consumers (Lee et al., 2020; Park et al., 2022; Vink et al., 2026). However, due to the high moisture content of chicken breast, it is easier to be deteriorated during storage caused by microbial and enzymatic activities, causing a shortened shelf life (Kong et al., 2023; Wang et al., 2021). To preserve the high quality of chicken breast in a long time, freezing and refrigeration techniques are commonly used to inhibit microbial growth and retard protein oxidation (Bai et al., 2023; Jin et al., 2026). At the beginning of further processing, the frozen chicken breast should be thawed to restore its quality to a state close to that of fresh chicken breast (Lee et al., 2026). Given that different thawing methods operate via distinct mechanisms, they significantly affect the physical characteristics, chemical characteristics and sensory quality of thawed chicken breast. Therefore, selecting an suitable thawing technology is essential for maintaining its high quality (Xie et al., 2025).

To achieve better thawing effect during the batch thawing process, it is important to improve both batch thawing uniformity and thawing efficiency (Gao et al., 2023). Water thawing (Wang et al., 2024) and air thawing (AT) (Li et al., 2020) require prolonged thawing times and are not suitable for rapidly processing batch frozen food (Çalışkan Koç et al., 2025). Although microwave thawing (Llave et al., 2020) and ultrasonic thawing (Ma et al., 2025) can achieve rapid thawing via polar molecular vibration and cavitation effects, they are limited by superficial microwave penetration depth and the rapid attenuation of ultrasonic waves, often causing localized overheating and compromising batch uniformity, which ultimately affects the quality of thawed food (Acheampong et al., 2025). In contrast, in terms of vacuum steam thawing (VST), the water vapor is introduced into a vacuum environment to thaw the frozen food rapidly and uniformly (Cai et al., 2018). Moreover, the vacuum environment effectively inhibits microbial growth, retards the oxidation of proteins and lipid, which helps preserve the quality of thawed food (Wang et al., 2022). To further optimize VST, our research group incorporated ice crystal sublimation into the thawing process and proposed a novel vacuum sublimation-rehydration thawing (VSRT). In terms of VSRT, the synergistic effect of low-pressure environment and heating plate heating promotes of the sublimation of ice crystal within frozen food, creating numerous tiny pores that facilitate water vapor diffusion and condensation heat exchange, thereby improving the thawing performance (Chen et al., 2022; Liu et al., 2023). Xue et al. (2025) confirmed in pork thawing experiments that VSRT exhibited the best overall performance compared with AT and VST. Thawing time was reduced by 55.37% and 34.61%, and color difference could be reduced by 87.04% and 82.76%, respectively.

The rehydration stage is one of the key stages in VSRT, and its main influencing factors include rehydration temperature and rehydration volume (Chen, 2022; Chen et al., 2023b). Li et al. (Li et al., 2024b) adopted the internal water vapor generation method, which involved directly introducing water into the vacuum chamber and utilizing low-pressure environment to cause boiling and vaporization. However, this method consumed a large amount of water, and the water used in thawing was prone to contamination by drips from frozen food and could not be recycled. To address these shortcomings, this study employed the external water vapor generation method. By applying the external water vapor generation module (EWVGM), a sufficient amount of water vapor was directly generated and then introduced into the vacuum chamber. The application of EWVGM simplified the influencing factor control in rehydration process, requiring only the adjustment of rehydration temperature. In this research, chicken breasts with initial temperatures of −18 °C were used as the research subjects (each approximately 200 g), with a total of 10 pieces. The aim was to find out the influences of various rehydration temperatures on thawing batch chicken breasts after applying the EWVGM in the thawing system, with the expectation of optimizing VSRT and providing theoretical guidance for the food processing industry.

## 2 Devices and principles of system

The VSRT involves sublimation stage and rehydration stage (Sun et al., 2025b). In the sublimation stage, the pressure inside vacuum chamber (P_IVC_) rapidly decreases. When the value of P_IVC_ is lower than that of ice crystal saturation pressure (125 Pa at −18 °C (Murphy & Koop, 2005)), the ice crystals sublimate, forming many tiny pores in the frozen food (Chen et al., 2020). In the rehydration stage, water vapor is generated by EWVGM. Then, it is introduced into the vacuum chamber, which causes the water vapor partial pressure (P_WVP_) inside the vacuum chamber to quickly increase. The P_WVP_ gradient between the frozen food interior and the vacuum chamber interior is created. This phenomenon drives the water vapor to diffuse to the frozen food for condensation heat exchange (location: tiny pores and surfaces of frozen food), thereby causing the ice crystals within frozen food to melt, ultimately completing the thawing process and simultaneously replenishing moisture to the frozen food (Kopec et al., 2022).

**Fig. 1:**
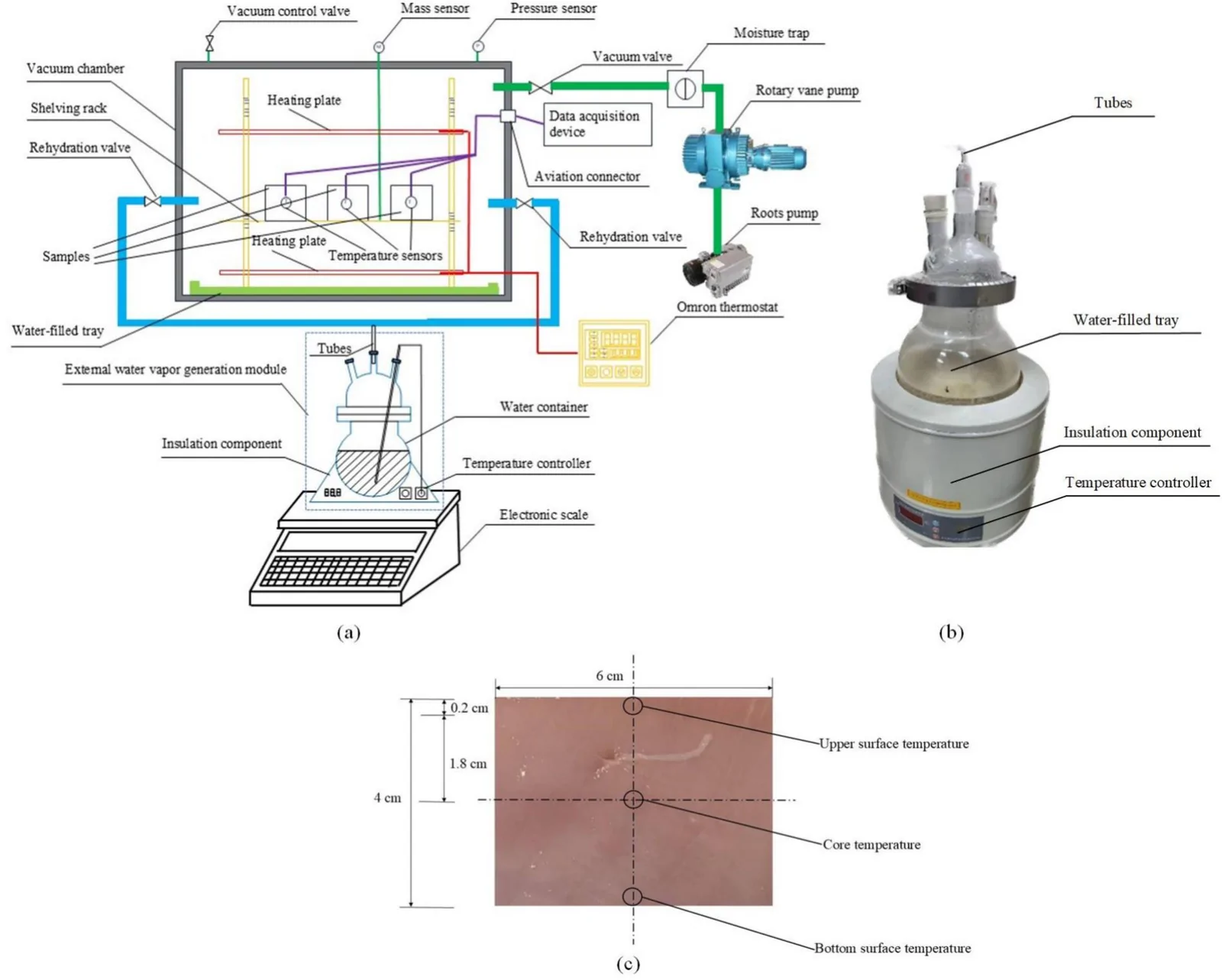
VSRT device & thermocouple arrangement; (a) VSRT device; (b) EWVGM; (c) Cross-section diagram of thermocouple arrangement in sample.

As shown in Fig. 1 (a, b), the VSRT device includes a vacuum chamber (the interior dimension of chamber was 600 mm × 460 mm × 400 mm), data acquisition equipment, rehydration equipment, heating equipment and vacuum equipment. The vacuum equipment comprises one vacuum pump set (Roots pump, rotary vane pump), one moisture trap, one vacuum control valve and one vacuum valve. By means of coordinating vacuum valve and vacuum pump set, the P_IVC_ can be controlled to the required range. The moisture trap is used to absorb moisture to avoid it from being introduced into the vacuum pump set and influencing the performance of vacuum pump set. The vacuum control valve can regulate P_IVC_. Heating equipment consists of an Omron thermostat, a shelving rack (the interior dimension of rack is 420 mm × 400 mm) and two heating plates (symmetrically arranged on both sides of frozen food, each 420 mm × 400 mm). Two heating plates serve as heat sources, with their temperatures controlled by Omron thermostat; the shelving rack is used to hold the frozen food. The rehydration equipment consists of an EWVGM (including tubes, a water container, an insulation component and a temperature controller) and two rehydration valves. The EWVGM is used to generate saturated water vapor, while two rehydration valves can control the on-off of the connection path between EWVGM and vacuum chamber. The data acquisition equipment comprises one mass sensor, one electronic scale (for measuring the water mass change in EWVGM), several T-type thermocouples (0.2 cm in diameter), a pressure sensor, one data acquisition device and one aviation connector (line connection). This equipment monitors and collects parameters during the thawing process. The specifications of equipment and instruments are presented in Table 1, and the accuracy of them was calibrated according to reference (Sun et al., 2026a) prior to the experiments.

**Table 1:** Equipment and instruments.

Equipment/ instruments	Model/type	Parameters	Accuracy
pH meter	Sigma Instruments (PH8180-0-00), China	0.00–14.00 pH	±0.05 pH
Omron thermostat	Omron (E5CC-800), Japan	20–180 °C	±0.5 °C
Pressure sensor	GE-Druck (UNIK5000), USA	0–100 kPa	±0.04% FS
Roots pump	Jiangyin Tiantian Vacuum Equipment Manufacturing Co., Ltd. (ZJP-70), China	Ultimate pressure: 0.05 Pa	±0.01 Pa
Rotary vane pump	Leybold SOGEVAC (SV40B), German	Ultimate pressure: 150 Pa	±1 Pa
Electronic scale	Wuyi Zhuheng Electronics Co., Ltd. (ACS—H7), China	0–30.0 kg	±0.1 g
Texture analyzer	Stable Micro Syste (TA-XT2i), UK	0.1–300 mm (test distance) 0–50 kg (test stress) 0.01–40 mm/s (test speed)	±0.001 mm ±0.1 g
Power meter	UNI-T (UT230E), China	0.001–9999 kWh	±1% Rdg
Temperature sensor	Omega (T-type), USA	−200–350 °C	±0.5 °C
Mass sensor	Jinnuo (JLBS-M2), China	0–3000 g	±0.5 g
Colorimeter	Konica Minolta (CR-400), Japan	0.01% ∼ 160% (reflectivity)	±6% FS
Water vapor generator	Beijing Yong Guangming Medical Instruments (SXTW), China	0–399 °C	±1°C

## 3 Methods and parameter analysis

### 3.1 Methods

#### 3.1.1 Pre-treatment of samples

1) The chicken breasts used were in the same batch. They were bought from a Shanghai market. Each chicken breast weighed 200 ± 3 g and was transported back to the laboratory within 30 min (preserved with ice cubes).

2) The initial parameters of fresh chicken breasts were measured. Additionally, chicken breasts of identical specifications were chosen for the experiment. Fresh chicken breasts were measured for mass, pH, lightness, redness, yellowness, hardness, elasticity, adhesiveness and cohesion to ensure they belonged to the same batch specification, thereby minimizing the interference of individual differences in the experiments. The parameter error margins for the same chicken breast batch were required to be within 5% (Junior et al., 2024). The mass of chicken breast was measured using an electronic scale. The detailed measurement methods for the other parameters were provided in Section 3.2. The chicken breast batch meeting the criteria of error margin was selected for subsequent processing and experiments. The parameters of the selected fresh chicken breasts were as follows: mass was 200 ± 3 g, pH was 6.05 ± 0.01, lightness was 52.48 ± 0.10, redness was 3.34 ± 0.11, yellowness was 5.79 ± 0.12, hardness was 1620.34 ± 38.21 g, elasticity was 0.76 ± 0.02, adhesiveness was 7.85 ± 0.03 g·s, and cohesion was 0.73 ± 0.01.

3) As shown in Fig. 1 (c), the thermocouples were inserted to the upper surface, bottom surface and core of each chicken breast. All the depths of them were 0.2 cm.

4) Based on the two standards (SC/T3101–2010 & NYT3524–2019), −18 °C (sample core temperature) was set as the initial thawing temperature; 4 °C (core temperature of fastest-thawing sample) was set as the final thawing temperature, meanwhile the other final sample core temperatures were required to be maintained at 0–4 °C.

5) All chicken breasts were placed in one freezer (−30 °C) for 5 h. Once the sample core temperatures were − 18 °C, some samples were prepared for conducting experiment (Zilio et al., 2018). The remaining samples were put in another freezer (−18 °C) and later taken out for subsequent experiments.

#### 3.1.2 VSRT experiment (external water vapor generation)

First, two heating plates were power on. The heating plate temperature was set via Omron thermostat. Once this temperature value stabilized, the samples were evenly distributed on the shelving rack. Next, the door of vacuum chamber was closed. Then, the vacuum pump set was operated to quickly decrease P_IVC_ to about 30 Pa, and the P_IVC_ was maintained at that level. When the parameter monitored by mass sensor indicated that samples achieved the required sublimation dehydration ratio (i.e., the ratio of sample mass lost during the sublimation stage to their total mass before thawing), the vacuum valve was immediately turned off and two rehydration valves were turned on. At this moment, P_IVC_ value was smaller than the water saturation pressure (rehydration temperature) in EWVGM. The water quickly boiled and generated water vapor, which was then transported to the vacuum chamber and diffused to tiny pores and the surface of samples driven by P_WVP_ gradient. The water vapor condensed and released latent heat at these locations. Upon absorbing the latent heat, the samples were heated and were ultimately thawed.

In light of the preliminary investigation of VSRT (Sun, Chen, et al., 2026; Sun, Wu, Xu, et al., 2025), the appropriate sublimation dehydration ratio (3%), heating-plate-to-frozen-food distance (80 mm), heating plate temperature (40 °C) and initial environmental temperature (20 °C) were determined and adopted for the experiments in this study. Meanwhile, a higher rehydration temperature was beneficial for accelerating the thawing process, but it should not exceed the denaturation temperature of chicken breast (56.31 °C) (Hu, 2023; Sun, Wu, et al., 2026), so the rehydration temperatures of 35, 40, 45, 50, and 55 °C were selected for comparative experiments. To decrease the effect of individual sample variability, all experiments were repeated for three times under each thawing condition.

#### 3.1.3 AT experiment

Chicken breasts were placed on the tray that was free from any heat source influence. The thawing was performed in air at 20 ± 1 °C (Zhang et al., 2021).

#### 3.1.4 VST experiment

Chicken breasts were evenly distributed on that shelving rack inside the vacuum chamber. Additionally, the P_IVC_ was set to 5.0 ± 0.1 kPa (Sun, Wu, & Ye, 2025). Then, two rehydration valves should be turned on. The saturated water vapor (the optimal rehydration temperature) was introduced into vacuum chamber. Water vapor heated samples via condensation heat exchange.

#### 3.1.5 VSRT experiment (internal water vapor generation)

In contrast to the VSRT experiment (external water vapor generation), this experiment only modified the rehydration method. The tube inside the water container was extended to be submerged in the water. After turning on the rehydration valves, the water (the optimal rehydration temperature obtained in the experiment in Section 3.1.2) was introduced into the water-filled tray below the heating plate because of the difference in pressure between the interior and exterior of vacuum chamber. Since the P_IVC_ was lower than the saturation pressure of water, the water quickly vaporized, generating saturated water vapor. All other experimental conditions were kept consistent with the optimal parameters used in the VSRT experiment (external water vapor generation). The rehydration volume was determined as the optimal value based on single-factor experiments following the method of Li et al. (Li, Wu, Chen, et al., 2024).

### 3.2 Evaluation indexes

#### 3.2.1 Thawing time & thawing rate

The thawing efficiency directly reflects the speed at which the sample core temperature rises. This evaluation index includes thawing rate and thawing time. Thawing time means the total time from the thawing beginning until all samples are completely thawed (Wang et al., 2020). Thawing rate follows the notion of “ice peak advance rate” from freezing to quantify the influence of sample shape on heat and mass transfer, and can be calculated using Formula (1) (Chen et al., 2020):

(1) $$v=\frac{D}{t_{r}}$$

where $t_{r}$ means the time from moment 1 (surface temperature is 0 °C) to moment 2 (core temperature is 4 °C), h; *D* means the minimum distance from core to surface, cm; *v* means thawing rate, cm/h.

#### 3.2.2 Batch thawing uniformity

The batch thawing uniformity can be characterized by the temperature coefficient of variation (TCOV). This parameter reflects the sample core temperature dispersion (Sun et al., 2022). A smaller TCOV indicates better batch thawing uniformity. The TCOV can be calculated using Formula (2):

(2) $$\text{TCOV}=\frac{1}{T_{a}}\sqrt{\frac{1}{n-1}\sum_{i=1}^{n}{\left(T_{i}-T_{a}\right)}^{2}}$$

where $T_{a}$ means average temperature among all temperature points, °C; $T_{i}$ is measurement point temperature, °C; n is measurement point number; TCOV is temperature coefficient of variation.

#### 3.2.3 Moisture change ratio (MCR)

The MCR can reflect the extent of moisture replenishment in frozen food during VSRT. A larger MCR indicates a greater rehydration effect (Wang et al., 2025). The MCR can be calculated by Formula (3):

(3) $$\mathrm{MCR}=\frac{M_{1}-M_{i}}{M_{0}}\times 100\%$$

where, *M*_1_ was sample mass after thawing, kg; *M*_i_ was sample mass after ice crystals sublimating, kg; *M*_0_ was sample mass prior to thawing, kg.

#### 3.2.4 Thawing loss

The thawing loss can assess juice loss after the samples are thawed (Yang et al., 2025), and it can be calculated using Formula (3) (averaging the measured values for each sample):

(3) $$\eta =\frac{M_{0}-M_{1}}{M_{0}}\times 100\%$$

where *η* represents thawing loss.

#### 3.2.5 Color parameters

The ∆E can evaluate the freshness of thawed chicken breast. This parameter is obtained using Formula (4) (averaging the measured values for each sample) (Li, Wu, Wang, et al., 2024). The yellowness (*b*), redness (*a*) and lightness (*L*) of samples are obtained by colorimeter.

(4) $$∆E=\sqrt{{\left(b^{\prime }-b^{\prime \prime }\right)}^{2}+{\left(a^{\prime }-a^{\prime \prime }\right)}^{2}+{\left(L^{\prime }-L^{\prime \prime }\right)}^{2}}$$

where ∆E means color difference; *L* means lightness; *a* means redness; *b* means yellowness; ${}^{\prime }$ means before thawing; ${}^{\prime \prime }$ means thawing completed.

#### 3.2.6 pH

The pH value can assess the spoilage and autolysis of thawed samples. It is determined as follows (Zhang et al., 2016): Weigh 10 g of minced chicken breast; thoroughly mix it evenly with deionized water (100 mL); allow the mixture to stand for 30 min, then filter it through filter paper; finally, measure the pH of filtrate with pH meter (averaging the measured values for each sample).

#### 3.2.7 Texture parameters

Texture parameters consist of hardness, cohesion, adhesiveness and elasticity. They directly reflect the texture characteristics of thawed chicken breast (Wu et al., 2025). In this study, a texture analyzer (P/50 probe) was used. The detailed method was as follows: Samples were cut into rectangular cuboids with dimensions of 20 mm by 20 mm by 10 mm. Set the pre-measurement speed to 3.00 mm/s, the mid-measurement speed to 1.00 mm/s, the post-measurement speed to 1.00 mm/s, the trigger force to 5 g, the compression distance to 10 mm, and the compression interval between two compressions to 5 s. The texture parameters for analysis were obtained from the average values of each sample.

#### 3.2.8 Specific energy consumption (SEC)

The SEC value can evaluate the energy utilization efficiency (Nattawut et al., 2025). It can be calculated using Formula (5):

(5) $$\psi =\frac{E_{i}}{M_{0}}$$

where ψ is SEC, which including total SEC, SEC for SEC for rehydration equipment (SEC-RE), SEC for heating equipment (SEC-HE) and vacuum equipment (SEC-VE), MJ/kg; $E_{i}$ means the energy consumption corresponding to SEC, MJ.

#### 3.2.9 Water consumption & water vapor utilization ratio

The water consumption can be calculated by reading the value shown on electronic scale placed beneath the EWVGM and using Formula (6). The water vapor utilization ratio (WVUR) can assess the ratio of water vapor supplemented into samples during the rehydration stage, and is calculated by Formula (7).

(6) $$M_{\text{water}}=M_{\mathrm{WVG}-0}-M_{\mathrm{WVG}-1}$$

(7) $${\mathrm{UR}}_{\mathrm{WV}-E}=\frac{M_{1}-M_{\mathrm{ICS}}}{M_{\text{water}}}\times 100\%$$

where $M_{\mathrm{water}}$ is water consumption of EWVGM, g; $M_{\mathrm{WVG}-0}$ is initial mass of EWVGM (including water), g; $M_{\mathrm{WVG}-1}$ is mass of EWVGM (including water) after thawing, g; ${\mathrm{UR}}_{\mathrm{WV}-E}$ is WVUR; $M_{\mathrm{ICS}}$ is sample mass after ice crystal sublimation, g.

### 3.3 Normalization evaluation

The normalized evaluation is adopted in this research to directly contrast the performance of VSRT under different thawing conditions (Wenlong et al., 2026). The score ranges from 0 (worst) to 1.00 (best). The scores are calculated using Formulas (8) and (9) (Sinsomboonthong, 2022):

(8) $$S_{H}=\frac{Y-Y_{\min}}{Y_{\max}-Y_{\min}}$$

(9) $$S_{L}=1-\frac{Y-Y_{\min}}{Y_{\max}-Y_{\min}}$$

where $S_{H}$ is the score (higher score indicates better performance); $S_{L}$ is the score (lower score indicates better performance); $Y_{\max}$ is the maximum value of evaluation index; $Y_{\min}$ is the minimum value of evaluation index.

### 3.4 Uncertainty analysis and statistical analysis

The absolute uncertainty can be calculated using Formula (10), which is applicable to directly measured parameters. The combined uncertainty is obtained using Formulas (11) and (12), and it is applicable to indirectly calculated evaluation indexes (Biffin et al., 2019). After calculation, the uncertainties of pH, ∆E, thawing loss, thawing rate, SEC, and WVUR were 0.12%, 0.23%, 0.14%, 0.12%, 0.06% and 0.14%, respectively. In this study, the SPSS software was used for statistical analysis. Significance differences were assessed using Duncan multiple-range test (*P* < 0.05).

(10) $$u=\frac{\mathrm{ac}}{\sqrt{3}}$$

(11) $$Y=f\left(x_{1},x_{2},∙∙∙x_{j}\right)$$

(12) $$u_{Y}={\left[\sum_{i=1}^{N}{\left(\frac{\mathrm{\partial Y}}{\partial x_{i}}\right)}^{2}u^{2}\left(x_{i}\right)\right]}^{\frac{1}{2}}$$

where ac means accuracy; u means uncertainty; $x_{i}$ is input variate; Y is evaluation index.

## 4 Results and discussion

### 4.1 Process of VSRT

**Fig. 2:**
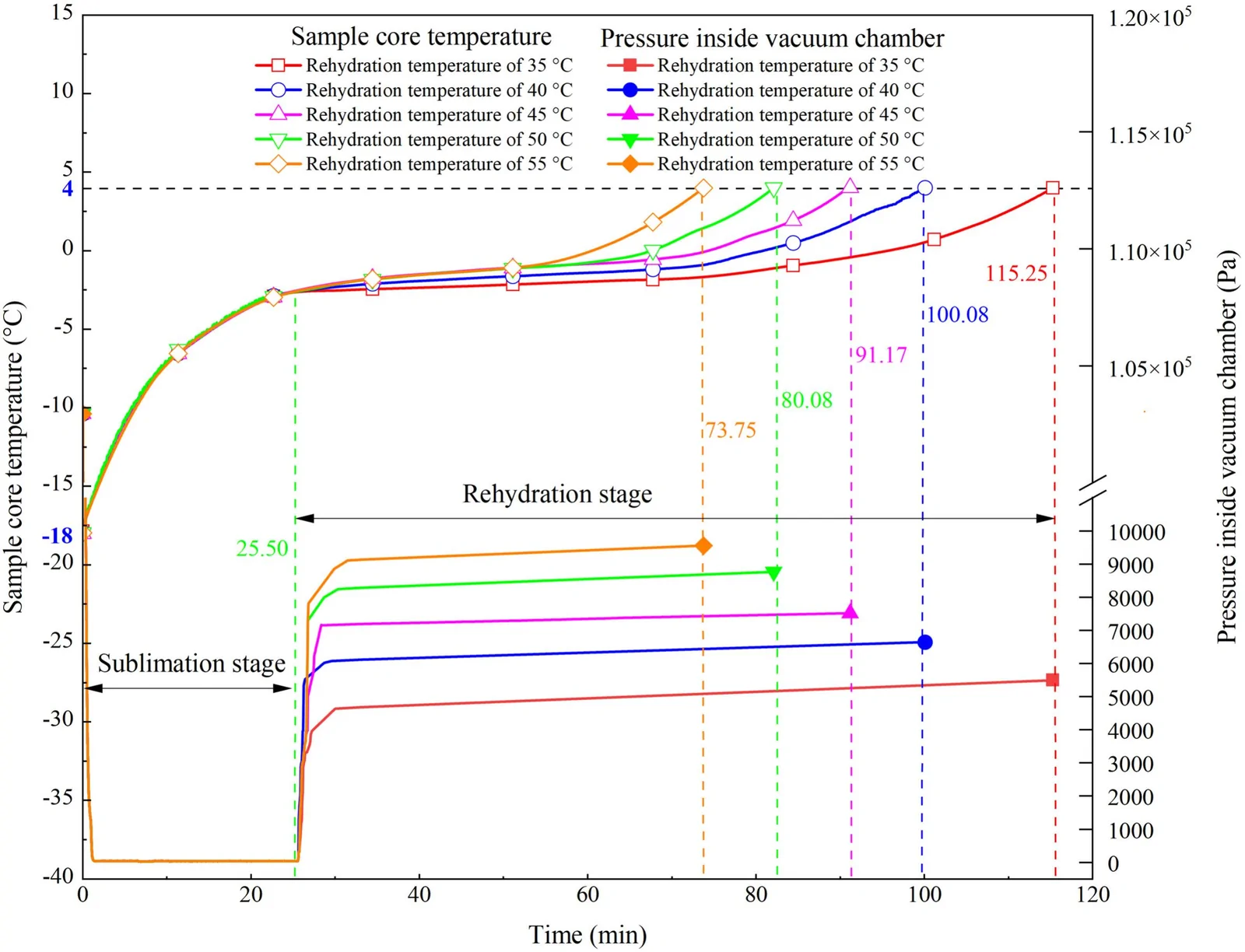
Thawing cures under different rehydration temperatures.

Various rehydration temperatures affect P_IVC_, and condensation and diffusion of water vapor, thereby affecting the process of VSRT. Thawing curves and the total sample mass changes under different rehydration temperatures are shown in Fig. 2 and Table 2, respectively.

**Table 2:** Total sample mass changes during VSRT (including sublimation stage and rehydration stage).

Rehydration temperature (°C)	Sublimation stage completed	Rehydration stage completed		
	M_i_ (g)	M_1_ (g)	M_1_-M_i_ (g)	MCR
35	1940.0	1967.6	27.6	1.38%
40	1940.0	1972.8	32.8	1.64%
45	1940.0	1974.8	34.8	1.74%
50	1940.0	1977.0	37.0	1.85%
55	1940.0	1970.4	30.4	1.52%

**Fig. 3:**
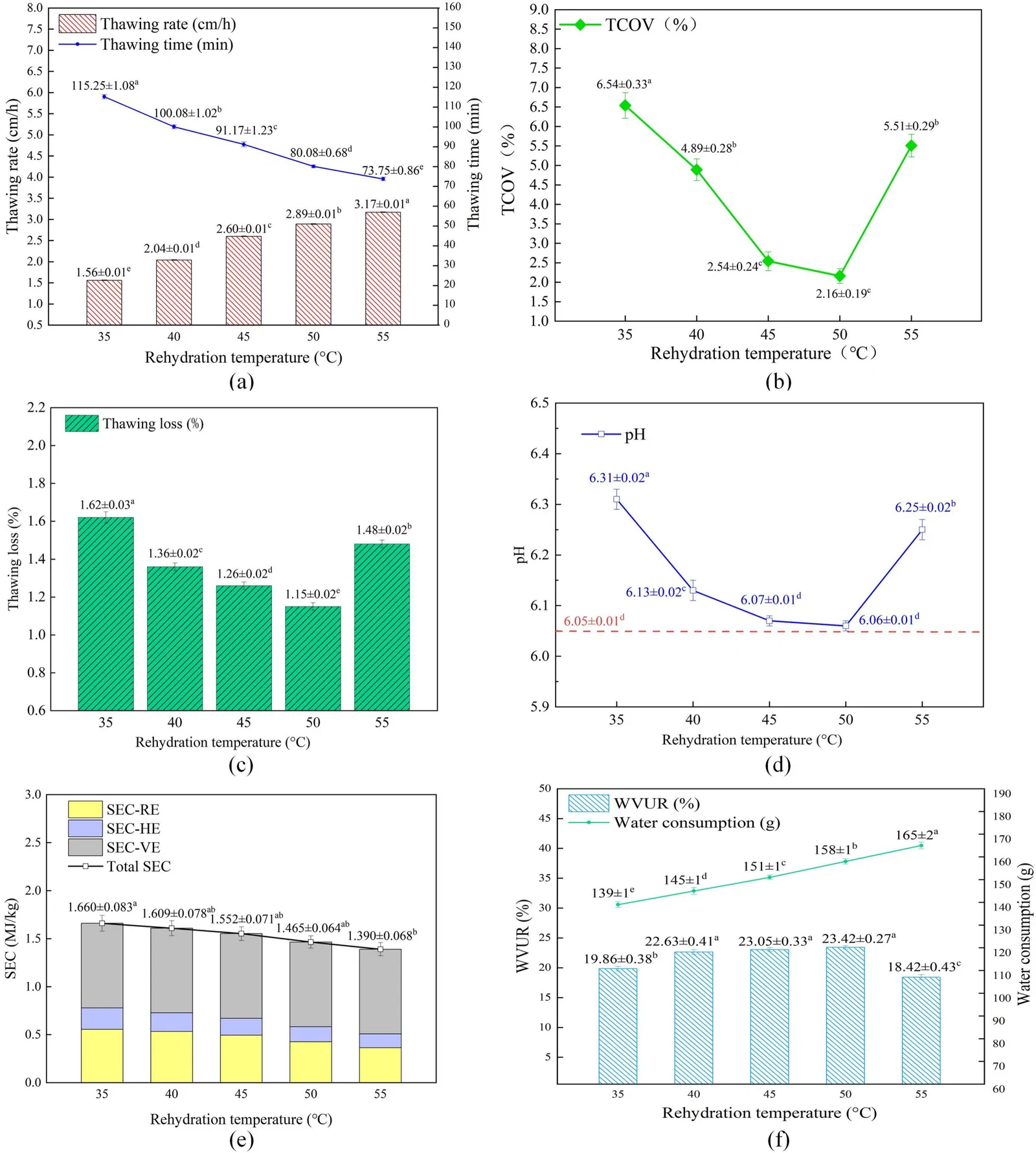
Variations of thawing performance under different rehydration temperatures; (a) thawing efficiency; (b) TCOV; (c) thawing loss; (d) pH; (e) SEC; (f) WVUR.

As presented in Fig. 2, in the sublimation stage, the P_IVC_ decreased rapidly and stabilized at about 30 Pa. Meanwhile, the sample core temperature generally showed an upward trend, but the heating rate of sample core temperature decreased after sample core temperature reached −5 °C. The heating rate was defined as the temperature change per unit minute, i.e., the first derivative of sample core temperature with respect to time during the thawing process, which corresponded to the slope of tangent line at any given time on the thawing curves shown in Fig. 3. The unit of heating rate was “°C/min”. The reason for this phenomenon was that the vacuum pump set continuously and efficiently operated during this stage, rapidly reducing the value of P_IVC_ to be less than the ice crystal saturation vapor pressure at −18 °C (125 Pa), thereby promoting the sublimation of ice crystal within chicken breasts (Sun et al., 2026b). This process resembled the ice crystal sublimation in freeze drying. The pressure of 30 Pa was far lower than the saturation vapor pressure of ice crystals at −18 °C (125 Pa), thereby satisfying the requirement for ice crystal sublimation (Tomas-Egea et al., 2018). Additionally, the incompletely frozen myofibrillar proteins and collagen in samples rapidly froze due to the rapid environmental pressure drop (Lee et al., 2024; Sun et al., 2026a). The latent heat released in this process, along with the heating plates, supplied heat to samples, driving the sample core temperatures to rise (Chen, Wu, Mao, et al., 2023). In addition, during the sublimation stage, the sample core temperature enhanced from −18 °C to −2.58 °C. Since the −5 ∼ −1 °C range was the maximum ice crystal melting zone (MICMZ) for chicken breasts (Du et al., 2025), more heat was necessary for melting ice crystals in the MICMZ. Thus, the core temperature increased faster within the −18 to −5 °C range, whereas the heating rate decreased in the −5 ∼ −2.58 °C range.

As shown in Fig. 2, during the rehydration stage, the P_IVC_ enhanced rapidly at first and then the enhancement rate of P_IVC_ became more slowly, while the sample core temperature increased slowly at first and then the increase rate of sample core temperature became more rapidly. The underlying mechanism of this phenomenon was as follows: After opening the rehydration valves, the EWVGM continuously introduced saturated water vapor into the vacuum chamber. Since the P_IVC_ was lower than the water vapor saturation pressure at that time, the overall pressure in VSRT device needed to be balanced, leading to the P_IVC_ to increase quickly. When the P_IVC_ approached the water vapor saturation pressure, the rising rate of P_IVC_ slowed down and gradually stabilized. During the process of rehydration, water vapor condensed at the tiny pores and surface of chicken breasts, releasing latent heat that was transferred to the cores of samples, driving the core temperature to gradually rise (Sun et al., 2025a). At the beginning of rehydration process, the sample core temperature was still within MICMZ, where more heat was required for melting ice crystals, making rapid heating difficult. After the sample core temperature passed through MICMZ, the heat required for samples to further heat up became mainly sensible heat, and the heating rate accelerated significantly until the samples were completely thawed (Sun et al., 2026b). Moreover, water vapor with higher rehydration temperature could provide more heat to release during condensation process, enabling the samples to pass through MICMZ more quickly, and a higher heating rate was obtained. This phenomenon was particularly obvious in the temperature range of −1–4 °C.

As illustrated in Table 2, since the heating plate temperature and the sublimation dehydration ratio remained constant, the total sample mass changes during the sublimation stage were the same. However, the MCR enhanced first and then declined with the enhancement of rehydration temperature. The maximum MCR (1.85%) occurred at a rehydration temperature of 50 °C. This was because appropriately increasing the rehydration temperature could increase temperature difference between water vapor and samples, thereby enhancing the heat and mass transfer, and promoting the moisture replenishment. However, an excessively high rehydration temperature (55 °C) caused a larger temperature gradient inside samples, reducing the uniformity of ice crystal melting in different parts of samples, easily causing partial collapse of tiny pores (Chrobak et al., 2026), hindering the efficient and continuous water vapor condensation, and resulting in a decrease in MCR.

### 4.2 Thawing efficiency & batch thawing uniformity

For batch thawing of chicken breasts, both greater batch thawing uniformity and higher thawing efficiency are required (Watanabe & Ando, 2021). The variations of the above-mentioned thawing performance under various rehydration temperatures are illustrated in Fig. 3 (a, b).

As presented in Fig. 3 (a,b), at rehydration temperatures of 35, 40, 45, 50 and 55 °C, the thawing times were 115.25, 100.08, 91.17, 80.08 and 73.75 min, respectively, showing a monotonically decreasing trend; the thawing rates were 1.56, 2.04, 2.60, 2.89 and 3.17 cm/h, respectively, showing a monotonically increasing trend; and the TCOVs were 6.54 × 10^−2^, 4.89 × 10^−2^, 2.54 × 10^−2^, 2.16 × 10^−2^, and 5.51 × 10^−2^, respectively, showing a trend of first declining then enhancing, with the minimum (best uniformity) at 50 °C. These changes could be attributed to the following. Increasing the rehydration temperature allowed water vapor to carry more heat, which was transferred to the sample cores via condensation heat exchange, thereby improving thawing efficiency. A moderate increase in rehydration temperature could enhance the heat and mass transfer efficiency, promote both fast diffusion and uniform condensation of water vapor with samples at different positions, and thereby improve the batch thawing uniformity. However, an excessively high rehydration temperature (55 °C) caused a larger temperature gradient inside samples, reducing the heat transfer uniformity (causing uneven melted water). When uneven melted water moved, it could compress and damage the muscle fiber structures of samples, which easily caused uneven ice crystal melting, triggering local collapse of tiny pores, hindering uniform condensation heat exchange, and ultimately decreasing batch thawing uniformity (Cai et al., 2020).

### 4.3 Thawing loss

Higher market value of chicken breast is attributed to better water retention of them, as reflected by lower thawing loss. (Martinek et al., 2022). The variation of thawing loss under different rehydration temperatures is illustrated in Fig. 3 (c).

As presented in Fig. 3 (c), when rehydration temperatures were 35, 40, 45, 50 and 55 °C, thawing losses were 1.62%, 1.36%, 1.26%, 1.15% and 1.48%, respectively. This parameter declined initially and then enhanced with the increasing rehydration temperature, and reached the optimal value (1.15%) at the rehydration temperature of 50 °C. This was because moderately increasing the rehydration temperature to 50 °C could decrease thawing time, dry loss and juice dripping loss caused by prolonged exposure of samples in the thawing environment (Chen et al., 2022). This drawback caused by an excessively long thawing time was also mentioned in the review by Leygonie et al. (2012). Meanwhile, the water vapor at this rehydration temperature could be quickly and uniformly transferred to each sample, undergoing uniform and efficient condensation heat exchange, which promoted quick and uniform ice crystal melting within chicken breasts, prevented sharper ice crystal morphology from piercing the cell structure, and thereby reduced juice loss (Li & Sun, 2002). However, too high a rehydration temperature decreased the batch thawing uniformity and ultimately caused uneven ice crystal melting, thereby exacerbating the degree of juice loss.

### 4.4 Color parameters

Good color retention of thawed samples means the freshness of them is higher (Ouyang et al., 2025).The variations of color parameters under different rehydration temperatures is shown in Table 3.

**Table 3:** Color parameters and texture parameters of samples.

Rehydration temperature /State	*b*	*a*	*L*	*ΔE*
Fresh chicken breast	5.79 ± 0.12^b^	3.34 ± 0.11^a^	52.48 ± 0.10^a^	–
35 °C	6.54 ± 0.14^a^	2.82 ± 0.13^b^	44.47 ± 0.12^e^	8.06 ± 0.23^a^
40 °C	6.41 ± 0.14^a^	2.97 ± 0.11^ab^	46.23 ± 0.13^c^	6.29 ± 0.22^b^
45 °C	6.32 ± 0.11^a^	3.02 ± 0.14^ab^	49.93 ± 0.12^b^	2.62 ± 0.21^c^
50 °C	5.92 ± 0.12^b^	3.15 ± 0.11^ab^	50.18 ± 0.13^b^	2.31 ± 0.21^c^
55 °C	6.48 ± 0.11^a^	2.93 ± 0.12^b^	45.56 ± 0.13^d^	6.97 ± 0.21^b^
Rehydration temperature /State	Cohesion	Adhesiveness (g·s)	Elasticity	Hardness (g)
Fresh chicken breast	0.73 ± 0.02^a^	7.85 ± 0.03^a^	0.76 ± 0.03^a^	1620.34 ± 38.21^c^
35 °C	0.64 ± 0.03^b^	7.64 ± 0.03^d^	0.61 ± 0.05^b^	1989.98 ± 45.51^a^
40 °C	0.68 ± 0.03^ab^	7.71 ± 0.02^bcd^	0.67 ± 0.04^ab^	1935.87 ± 42.76^ab^
45 °C	0.70 ± 0.02^ab^	7.74 ± 0.02^bc^	0.72 ± 0.02^ab^	1891.33 ± 35.98^ab^
50 °C	0.71 ± 0.02^ab^	7.79 ± 0.02^ab^	0.74 ± 0.02^a^	1832.73 ± 33.23^b^
55 °C	0.66 ± 0.03^ab^	7.67 ± 0.03^cd^	0.65 ± 0.03^ab^	1965.82 ± 44.89^ab^

Note: different superscripts in a column represent significant differences (*P* < 0.05).

As illustrated in Table 3, with the increase of rehydration temperature, the changes in the lightness (∆L), redness (∆a), yellowness (∆b), and *ΔE* o all initially decreased and then increased, obtaining their optimal values at a rehydration temperature of 50 °C (∆L = 2.30, ∆a = 0.19, ∆b = 0.13,*ΔE* = 2.31). This was because the loss of juice caused proteins and lipids to be more easily exposed to oxidase, resulting in oxidation and subsequent color changes (Carvalho et al., 2017). A moderate enhancement in rehydration temperature to 50 °C promoted heat and mass transfer, enabling water vapor to condense and exchange heat with chicken breasts quickly and uniformly, thereby accelerating ice crystal melting, minimizing cell damage from sharp ice crystals, and reducing juice leakage and component exposure. However, when the rehydration temperature was too high (55 °C), uneven melting of ice crystals formed sharp crystals that punctured cell membranes, aggravating the denaturation and oxidation of protein and lipid, and ultimately leading to significant deterioration of color (Du et al., 2022).

### 4.5 pH

A smaller pH change (ΔpH) of thawed sample indicates better quality retention of chicken breast (Huang et al., 2025). In this study, the pH of fresh chicken breast was 6.05, falling within the pH range of 5.70–6.10 specified by the standard of NY/T 2793–2015. The variation of pH under different rehydration temperatures is presented in Fig. 3 (d).

According to Fig. 3 (d), at rehydration temperatures of 35, 40, 45, 50 and 55 °C, the pH values of samples were 6.31, 6.13, 6.07, 6.06 and 6.25, respectively. When rehydration temperature increased, the pH values of samples first declined and then enhanced, achieving the minimum (6.06) at a rehydration temperature of 50 °C. Under this thawing condition, the ΔpH was the smallest (0.01). This trend of pH change was because the rapid and uniform thawing process enabled the ice crystals to melt quickly and uniformly, thereby reducing juice loss, inhibiting the decomposition of amino acids and proteins, and preventing local accumulation of alkaline substances (Zhou et al., 2019). Inhibiting the accumulation of alkaline substances could prevent the pH values of samples from rising too much (Qiao et al., 2026). Moderately increasing the rehydration temperature to 50 °C not only effectively reduced the thawing time but also achieved the best batch thawing uniformity, which delayed the spoilage and oxidation process of samples, and thus inhibited the increase in pH value.

### 4.6 Texture parameters

Texture parameters are key indicators for assessing the quality of thawed samples. A smaller variation in the texture parameters indicates less deterioration in texture (Mengzi et al., 2026). The variations of texture parameters under different rehydration temperatures are shown in Table 3.

As presented in Table 3, as the rehydration temperature increased, the variation range of texture parameters showed a trend of first rising and then dropping. The optimal values of hardness, elasticity, adhesiveness and cohesion were all achieved when the rehydration temperature was 50 °C. This was because when the rehydration temperature was 50 °C, the batch thawing uniformity was the best and the thawing time was shorter, which promoted the quick and uniform ice crystal melting, thus substantially reducing the losses of moisture and soluble proteins, and maximizing the maintenance of the original muscle tissue structure of samples (Sun et al., 2026a). This was consistent with the proposal of Bedane et al. (2018) that increasing thawing rate and improving thawing uniformity helped maintain the textures of chicken breasts. Under this thawing condition, the changes in cohesion, adhesiveness, elasticity and hardness of thawed chicken breast were the smallest compared to those of fresh chicken breast. Thus, under the thawing condition of a rehydration temperature of 50 °C, the texture of sample was the best maintained, which was more conducive to subsequent processing and handling.

### 4.7 SEC

The overall SEC is made up of SEC-RE, SEC-HE, and SEC-VE. A lower total SEC indicates greater energy utilization efficiency of VSRT device (Sun et al., 2026a). The variation of SEC under different rehydration temperatures is shown in Fig. 3 (e).

As shown in Fig. 3 (e), when the rehydration temperatures were 35, 40, 45, 50 and 55 °C, the total SEC values were 1.660, 1.609, 1.552, 1.465 and 1.390 MJ/kg, respectively. With the increase of rehydration temperature, the total SEC gradually decreased. This trend was because of three factors. First, the sublimation dehydration ratio remained constant under all thawing conditions, thus the energy consumption by vacuum equipment was also constant (Sun et al., 2026b). Secondly, the heating plate temperature remained constant under all thawing conditions, so the energy consumption of heating equipment was positively correlated with thawing time (Sun et al., 2025a). Finally, the energy consumption of rehydration equipment mainly came from heating water to the set rehydration temperature and maintaining it during rehydration stage. The process of heating water with EWVGM lasted only about 10 min, whereas the heat preservation process of EWVGM ran throughout the rehydration stage. Furthermore, the heat preservation process primarily relied on the insulation component, which required smaller amount of heat to maintain the temperature of water in EWVGM. Consequently, the rehydration time played a dominant role in the SEC-RE, but the values of SEC-RE were lower than those of SEC-VE. Considering the change in the above three SEC, the total SEC gradually decreased as the rehydration temperature increased.

### 4.8 Water consumption & water vapor utilization ratio (WVUR)

In addition to electric energy consumption, VSRT also entails water consumption for generating water vapor (Sun et al., 2026b). The WVUR is a direct metric for assessing water utilization efficiency. The variations of water consumption and WVUR under different rehydration temperatures are illustrated in Fig. 3 (f).

As presented in Fig. 3 (f), when the rehydration temperatures were 35, 40, 45, 50 and 55 °C, the water consumption values were 139, 145, 151, 158 and 165 g, respectively, and the WVUR values were 19.86%, 22.63%, 23.05%, 23.42% and 18.42%, respectively. As the rehydration temperature increased, the water consumption gradually increased, while the WVUR initially enhanced and then declined, and reached its optimal value (23.42%) at a rehydration temperature of 50 °C. This upward trend was attributed to the fact that higher temperatures corresponded to higher saturated water vapor pressures, facilitating the rapid vaporization of water in a low-pressure environment, thus resulting in an increase in water consumption (Chen, Wu, Wang, et al., 2023). Additionally, the water vapor with a higher temperature carried more heat, and thus had a higher efficiency in heat and mass transfer (Augusto et al., 2013). Moderately raising the rehydration temperature to 50 °C enhanced the condensation efficiency of water vapor, thereby reducing the water vapor loss that diffused to the inner surface of vacuum chamber. However, once the rehydration temperature was excessively high (55 °C), the tiny pores in samples tended to collapse, which hindered the efficient condensation of water vapor, resulting in a decreased WVUR.

### 4.9 Comprehensive evaluation

**Fig. 4:**
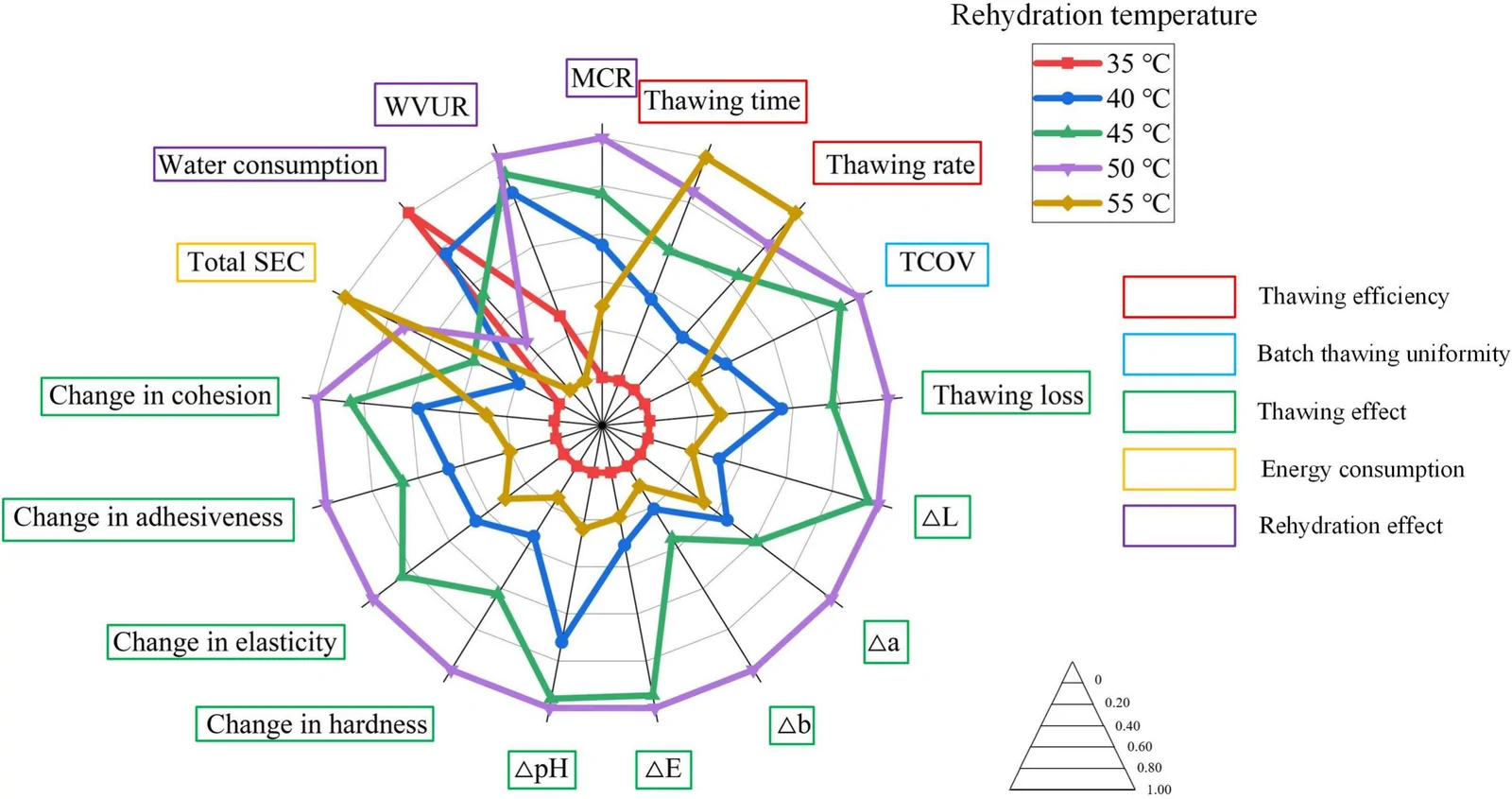
Comprehensive evaluation radar chart.

To comprehensively evaluate the overall impact of various rehydration temperatures on VSRT of batch chicken breasts, a comprehensive evaluation radar chart is presented in this paper, as illustrated in Fig. 4.

According to Fig. 4, 50 °C was the optimal rehydration temperature. Under this thawing condition, the thawing efficiency was higher, the batch thawing uniformity was the best, the thawing effect was the best (with the smallest ΔpH, the optimal color, the smallest changes in texture parameters and the smallest ΔpH), the total SEC was lower, and the rehydration effect was the best (with the highest MCR, the highest WVUR, and only a small increase in water consumption). Therefore, at a rehydration temperature of 50 °C, efficient and energy-saving batch thawing of chicken breast could be achieved.

### 4.10 Comparison of various thawing methods

To investigate the differences between VSRT (external water vapor generation) at the optimal rehydration temperature and other thawing methods, this paper compared it with AT, VST, and VSRT (internal water vapor generation). Following the single-factor experimental method described in Section 3.1.5, which only changed the rehydration volume while keeping other influencing factors constant, the rehydration volume (i.e., water consumption) under the optimal thawing conditions for VSRT (internal water vapor generation) was determined to be 1500 g of water. The differences in evaluation indexes among the different thawing methods are shown in Table 4.

**Table 4:** The performance of various thawing methods.

Thawing method/State		Thawing rate (cm/h)	Thawing time (min)	Thawing loss (%)	TCOV (×10^−2^)	MCR (%)
Fresh chicken breast		–	–	–	–	–
VSRT (external water vapor generation)		2.89 ± 0.01^a^	80.08 ± 0.68^d^	1.15 ± 0.02^d^	2.16 ± 0.19^c^	1.85 ± 0.02^a^
AT		0.61 ± 0.05^d^	249.17 ± 2.76^a^	6.11 ± 0.04^a^	9.92 ± 0.41^a^	–
VST (external water vapor generation)		1.41 ± 0.03^c^	146.75 ± 1.84^b^	2.91 ± 0.03^b^	7.95 ± 0.27^b^	–
VSRT (internal water vapor generation)		2.49 ± 0.02^b^	93.17 ± 1.08^c^	1.29 ± 0.02^c^	2.77 ± 0.21^c^	1.71 ± 0.02^b^
Thawing method/State	pH	*b*	*a*	*L*	*ΔE*	Total SEC (MJ/kg)
Fresh chicken breast	6.05 ± 0.01^d^	5.79 ± 0.12^c^	3.34 ± 0.11^a^	52.48 ± 0.10^a^	–	–
VSRT (external water vapor generation)	6.06 ± 0.01^cd^	5.92 ± 0.12^c^	3.15 ± 0.11^a^	50.18 ± 0.13^b^	2.31 ± 0.21^d^	1.465 ± 0.064^b^
AT	6.83 ± 0.01^a^	7.84 ± 0.14^a^	1.26 ± 0.21^b^	41.53 ± 0.17^e^	11.33 ± 0.30^a^	0^c^
VST (external water vapor generation)	6.55 ± 0.01^b^	7.61 ± 0.18^a^	1.71 ± 0.15^b^	43.12 ± 0.14^d^	9.67 ± 0.27^b^	7.536 ± 0.088^a^
VSRT (internal water vapor generation)	6.09 ± 0.01^c^	6.38 ± 0.12^b^	2.99 ± 0.13^a^	49.12 ± 0.14^c^	3.43 ± 0.23^c^	1.576 ± 0.073^b^
Thawing method/State	Water consumption (g)	WVUR (%)	Cohesion	Adhesiveness (g·s)	Elasticity	Hardness (g)
Fresh chicken breast	–	–	0.73 ± 0.01^a^	7.85 ± 0.03^a^	0.76 ± 0.02^a^	1620.34 ± 38.21^d^
VSRT (external water vapor generation)	158 ± 1^c^	23.42 ± 0.27^a^	0.71 ± 0.02^a^	7.79 ± 0.02^ab^	0.74 ± 0.02^a^	1832.73 ± 33.23^c^
AT	0^d^	–	0.54 ± 0.02^c^	7.36 ± 0.03^d^	0.58 ± 0.06^b^	2510.63 ± 96.64^a^
VST (external water vapor generation)	372 ± 2^b^	–	0.65 ± 0.03^b^	7.61 ± 0.03^c^	0.68 ± 0.05^ab^	2229.65 ± 67.54^b^
VSRT (internal water vapor generation)	1500 ± 1^a^	2.28 ± 0.21^b^	0.69 ± 0.02^ab^	7.72 ± 0.02^b^	0.69 ± 0.03^a^	1908.34 ± 36.21^c^

Note: Different superscripts in a column represent significant differences (*P* < 0.05).

As presented in Table 4, compared with AT and VST, VSRT exhibited significant advantages in batch thawing uniformity and thawing efficiency. It reduced the TCOV by 78.23% and 72.83%, and shortened the thawing time by 67.86% and 45.43%, respectively. Meanwhile, VSRT was better able to maintain the sample quality, as evidenced by the lowest thawing loss, the smallest changes in texture parameters, the best color and smallest ΔpH. Moreover, compared with VST, the VSRT could reduce the total SEC by 80.55% and saved 57.53% of water consumption. These differences were mainly attributable to the different thawing mechanisms: VSRT formed numerous tiny pores through ice crystal sublimation in samples, and combined with a higher rehydration temperature to enhance heat and mass transfer, promoting uniform and efficient condensation heat exchange between samples and water vapor, thus obtaining rapid and uniform thawing. In terms of AT, the air thermal conductivity was lower and this thawing method lacked forced convection, which caused slow and uneven heat transfer (Lee et al., 2021). VST lacked the tiny pores generated by ice crystal sublimation, leading to relatively lower condensation efficiency and correspondingly increased water consumption; it also required the continuous operation of vacuum pump set, causing an increase in the total SEC (Sun et al., 2025b).

Compared with the internal water vapor generation, the external water vapor generation exhibited significant advantages: it shortened thawing time by 14.05%, reduced TCOV by 22.02%, and decreased total SEC by 7.04%; additionally, the samples thawed with external water vapor generation exhibited superior quality in terms of color, pH, thawing loss and other parameters, compared with those thawed with internal water vapor generation. Moreover, the external water vapor generation utilized water more efficiently: its water consumption could be reduced by 89.47%, and the WVUR could be increased by 9.27 times, compared with those parameters obtained with internal water vapor generation. This was because water vaporization required heat absorption. The external generation occurred in the EWVGM, thus avoiding the heat loss from vacuum chamber. In contrast, the internal water vapor generation required water to absorb some heat from the vacuum chamber, which reduced the efficiency of heat and mass transfer in vacuum chamber, thereby slowing down the thawing process and hindering rapid and uniform diffusion of water vapor, ultimately decreasing the thawing effect and increasing the total SEC. Furthermore, for internal water vapor generation, the water introduced into vacuum chamber was easily contaminated by dripping juice from samples and could not be recycled. In contrast, the external water vapor generation introduced only water vapor into vacuum chamber, and the large amount of unvaporized water remained uncontaminated and could be recycled, thus effectively saving water consumption.

## 5 Conclusions

1) Increasing the rehydration temperature enhanced heat and mass transfer, enabling rapid and uniform transfer of water vapor to tiny pores and surface of chicken breast, thereby promoting the uniform and rapid ice crystal melting. However, once the rehydration temperature was excessively high, it caused partial collapse of tiny pore structures, which hindered water vapor condensation.

2) The optimal rehydration temperature was 50 °C, at which the best batch thawing uniformity (TCOV of 2.16 × 10^−2^), the lowest thawing loss (1.15%), the shortest thawing time (80.08 min), the best color (*ΔE* = 2.31), the smallest ΔpH (0.01), the best texture parameters, lower total SEC (1.465 MJ/kg), the highest WCR (1.85%) and the highest WVUR (23.42%) were achieved.

3) Compared with AT and VST, VSRT shortened thawing time by 67.86% and 45.43%, and reduced TCOV by 78.23% and 72.83%, respectively. Moreover, compared with VST, VSRT activated the vacuum pump set only in the sublimation stage, which reduced the total SEC by 80.55%. Furthermore, by adopting the EWVGM, VSRT saved 57.53% of water consumption compared with that in VST. Thereby, VSRT could save more electrical energy and water resources.

4) Compared with the internal water vapor generation method, the external water vapor generation method applied in VSRT reduced the TCOV by 22.02%, shortened the thawing time by 14.05%, decreased the total SEC by 7.04%, and obtained the best quality indicators in thawed chicken breast. Moreover, in terms of water consumption and WVUR, the EWVGM prevented the unvaporized water from being contaminated, allowing it to be recycled. Compared with the internal water vapor generation method, the external water vapor generation method reduced water consumption by 89.47% and increased the WVUR by 9.27 times, thus achieving efficient utilization of water.

## CRediT authorship contribution statement

**Yuyao Sun:** Writing – original draft, Investigation, Formal analysis. **Shanshan Chen:** Funding acquisition. **Weidong Wu:** Writing – review & editing, Funding acquisition. **Nating Xu:** Conceptualization. **Fangran Liu:** Project administration. **Anyuan Xue:** Methodology.

## Funding

This work was supported by the National Natural Science Foundation of China (NSFC) (No. 52176016) and Ningbo Municipal Public Welfare Research plan (2024S048).

## Declaration of competing interest

The authors declare that they have no known competing financial interests or personal relationships that could have appeared to influence the work reported in this paper.

## Data Availability

Data will be made available on request.
